# The Effects of an E-Mental Health Program and Job Coaching on the Risk of Major Depression and Productivity in Canadian Male Workers: Protocol for a Randomized Controlled Trial

**DOI:** 10.2196/resprot.6350

**Published:** 2016-11-15

**Authors:** JianLi Wang, Scott B Patten, Raymond W Lam, Mark Attridge, Kendall Ho, Norbert Schmitz, Alain Marchand, Bonnie M Lashewicz

**Affiliations:** ^1^ Department of Psychiatry University of Calgary Calgary, AB Canada; ^2^ Department of Community Health Sciences University of Calgary Calgary, AB Canada; ^3^ Mathison Centre for Mental Health & Education Hotchkiss Brain Institute University of Calgary Calgary, AB Canada; ^4^ Department of Psychiatry University of British Columbia Vancouver, BC Canada; ^5^ Attridge Consulting Inc. Minneapolis, MN United States; ^6^ Department of Emergency Medicine University of British Columbia Vancouver, BC Canada; ^7^ Digital Emergency Medicine University of British Columbia Vancouver, BC Canada; ^8^ Department of Psychiatry McGill University Montreal, QC Canada; ^9^ School of Industrial Relations University of Montreal Montreal, QC Canada

**Keywords:** Internet, RCT, men, workplace, major depression, prevention

## Abstract

**Background:**

Major depression (MDE) is prevalent in men and affects men’s health and productivity. Because of the stigma against depression and social/gender norms, men are less likely to seek help for emotion and stress-related issues. Therefore, innovative solutions tailored for men are needed. With rapid development of the Internet and information technologies, one promising solution that has drawn considerable attentions is electronic mental (e-mental) health programs and services.

**Objective:**

The objective of our study is to evaluate the effectiveness of the e-mental health program BroHealth on reducing the risk of having MDE and improving productivity and return to investment.

**Methods:**

The target population is Canadian working men who are at high risk of having MDE (N=1200). Participants will be recruited using the method of random digit dialing across the country and workplace advertisement. Eligible participants will be randomly allocated into the following groups: (1) a control group, (2) a group receiving BroHealth only, and (3) a group receiving BroHealth and telephone-based job coaching service. The groups will be assessed at 6 and 12 months after randomization. The primary outcome is the risk proportion of MDE over 12 months, which will be assessed by the World Health Organization's (WHO’s) Composite International Diagnostic Interview-Short Form for Major Depression. Intention-to-treat principle will be used in the analysis. The 12-month proportions of MDE in the groups will be estimated and compared. Logistic regression modeling will be used to examine the effect of the intervention on the outcome, controlling for the effects of baseline confounders.

**Results:**

It is anticipated that the randomized controlled trial (RCT) will be completed by 2018. This study has been approved by the Conjoint Health Research Ethics Review Board of the University of Calgary. The trial is funded by a team grant from the Movember Foundation, a global charity for men’s health. BroHealth was developed at the Digital Emergency Medicine, University of British Columbia, and the usability testing has been completed.

**Conclusions:**

BroHealth was developed based on men’s needs. We hypothesized that BroHealth will be an effective, acceptable, and sustainable product for early prevention of MDE in workplaces.

**ClinicalTrial:**

Clinicaltrials.gov NCT02777112; https://clinicaltrials.gov/ct2/show/NCT02777112 (Archived by WebCite at http://www.webcitation.org/6lbOQpiCG)

## Introduction

### Background

Major depression (MDE) affects workers’ health and productivity. In the United States, workers with depression cost an estimated US $44.01 billion per year in lost productivity [1]. One of the severe consequences of having MDE is suicide and Canadian national data showed that 76% of all suicides in 2009 were male [2]. Compounding men’s risk, men are less likely than women to seek help and to disclose depressive symptoms and often delay help seeking until symptoms become severe. If they do seek help, men are often worried about anonymity and prefer flexible and timely access. Men are socialized to be emotionally stoic and exemplify traditional masculine characteristics such as independence, self-reliance, and dominance [3]. Men are concerned over the perceived negative judgments from family and friends if they access treatment for depression. These gender-specific experiences along with a limited knowledge base about effective interventions call for innovative solutions tailored for men. With rapid development of the Internet and information technologies, one promising solution that has drawn considerable attentions is electronic mental (e-mental) health programs and services.

E-mental health is “the use of information and communication technologies to support and improve mental health, including the use of online resources, social media, and smartphone applications” [4]. E-mental health programs offer information about mental health or therapeutic services remotely through the Internet or by telephone. The increasing availability of fast broadband access provide e-mental health programs several advantages including easy and flexible access to services in remote areas and low costs. To a certain extent, e-mental health programs are closely aligned with men’s needs in terms of privacy protection, flexible, and timely access to help. There have been a number of e-mental health programs for therapeutic intervention. A recent review by Christensen and Petrie showed that by 2013 there had been 62 Web-based mental health interventions and 11 mobile apps [5]. Lal and Adair found 91 peer-reviewed publications on the application of e-mental health interventions between 2000 and 2010 [6]. However, the number of randomized controlled trials (RCT) has not been proportionate to the number of e-mental health programs. For example, only 30.1% of the 73 programs identified in Christensen and Petrie's review had been evaluated by one or more RCTs [5]. Many of the existing e-mental health programs for depression were developed based on the approach of cognitive behavioral therapy (CBT). The most widely disseminated e-mental health program based on CBT is MoodGYM developed in Australia [7]. Other well-known e-mental health programs for depression that have been evaluated by at least one RCT include Sadness [8], e-couch [9], BluePages [7], and myCompass [10]. Although e-mental health programs offer a non-threatening, convenient, and anonymous environment, some users reported that going through the CBT sessions and exercises were laborious, the sessions were not relevant with the users’ unique personal issues, and there was a lack of personal interaction [11]. Our research project, BroMatters, intends to overcome these limitations.

BroMatters is a project aiming to develop and evaluate an e-mental health program for early prevention of major depression in Canadian male workers who do not have MDE, but are at high risk. It is a collaboration among researchers and stakeholders from five Canadian universities and six national and local non-government organizations (NGOs). To develop the e-mental health program, BroMatters conducted a national survey in male workers who did not have MDE, but were at high risk, to understand their preferences of design features of e-mental health programs [12]. Informed by the survey results, the BroMatters team developed an e-mental health program (BroHealth) to be evaluated by a RCT.

### Objectives and Hypotheses

The primary objective of the proposed RCT is to evaluate the impacts of BroHealth and telephone-based job coaching on the risk of MDE among Canadian male workers who are at high risk of having MDE. We hypothesized that the 12-month risk proportion of MDE in the intervention group will be lower than that in the control group.

The secondary objectives of the proposed RCT are to (1) evaluate the impacts of BroHealth and telephone-based job coaching on changes in depression, anxiety, absenteeism and presenteeism, return on investment, and predicted MDE risk; and (2) compare participants who receive BroHealth only and those who receive both BroHealth and telephone-based job coaching services in changes in depression, anxiety, absenteeism and presenteeism, return on investment, and predicted MDE risk.

## Methods

### Study Design

The proposed mixed-methods study is a prospective, intention-to-treat, RCT with the following 3 arms: (1) control group (n=400) that will receive general information about men’s mental health (the Movember Foundation website on men’s mental health), (2) intervention arm 1 group (n=400) that will receive the e-mental health program, and (3) intervention arm 2 group (n=400) that will receive the e-mental health program, and telephone-based interactive work-life coaching.

**Figure 1:**
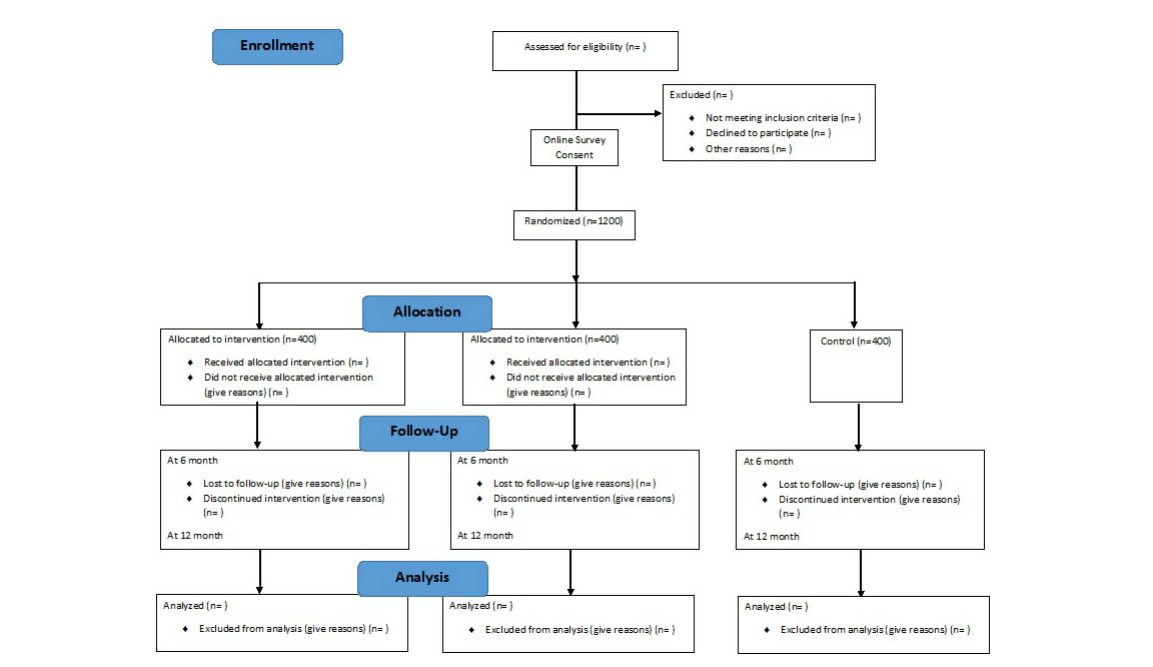
BroMatters clinical trial flow diagram.

An embedded qualitative interview component will be conducted with a sub sample of participants from the intervention groups to obtain in-depth perspectives about the effectiveness of interventions at the 12-month endpoint. A flow chart of the proposed trial based on the Consolidated Standards of Reporting Trials (CONSORT) criteria is shown in Figure 1.

### Study Setting and Inclusion Criteria

Eligible participants will be recruited from 10 provinces of Canada. Recruitment and fully structured baseline interviews will be conducted by Leger, a Canadian-owned polling and research firm; follow-up interviews will be conducted at the telephone interview laboratory of the Mathison Centre for Mental Health and Education, University of Calgary. The proposed trial will target Canadian male workers who are aged 18 years or older, are working for pay at the time of recruitment, and at the time of recruitment and baseline assessment, are not experiencing a MDE, but have a high risk of having MDE based on our prediction algorithm. Since it was found that 20% of Canadian adult men had a probability of 6.51% or higher in terms of the predicted MDE risk [13], for the proposed study, a personalized risk (or probability) of 6.51% or higher will be defined as high risk for men. In addition, the trial will target workers who may have had MDE in the past 12 months, but are in remission for at least 2 months prior to the study, have no language barriers to English or French, have access to the Internet for personal use, and can provide email and mailing addresses.

### Intervention

#### BroHealth

BroHealth is an e-mental health program which was developed to reduce men’s risk of having MDE and to improve productivity. The program was designed to be used by working men who don’t have MDE, but are at high risk determined by a multivariable risk prediction algorithm [13]. The development of BroHealth was informed by the results of a national survey in the target population, about high risk men’s preferences for the design features of e-mental health programs [12]. The product was developed at the Digital Emergency Medicine of the University of British Columbia, guided by a committee consisting of research team members with expertise in psychiatry, epidemiology, e-health, occupational psychology, addiction, information technology, and software programming. Before finalization, BroHealth was pilot tested among remaining team members, Stakeholder Advisory Committee members, and the general public recruited through personal networks and social media.

BroHealth contains the following key modules: (1) Information, (2) Self-Help, (3) Self-Check, and (4) Goal Setting and Tracking. The contents of each module are described in Textbox 1.

**The contents of each BroHealth module.**

Module and content

1. Information: information about stress, depression, depression in the workplace, and alcohol use in the workplace.

2. Self-Help: a cognitive behavior therapy (CBT) program (Living Life to the Full) developed in the United Kingdom, a mindfulness relaxation program, and specific strategies for dealing with work related issues.

3. Self-Check: online instruments for monitoring current depressive and anxiety symptoms, occupational functioning, and future risk of major depression (MDE).

4. Goal Setting: the S.M.A.R.T goal setting and tracking system.

BroHealth is a Web-based program that is, at the current stage, opened to research participants only. Participants can log in to BroHealth with a user name and password generated at the Digital Emergency Medicine of University of British Columbia. The novelty of BroHealth is that it was designed in a modular format by which users may select any module/section, without particular sequence. Based on the preferences of the target population and feedback from stakeholders, the contents of the program are balanced with text description and video clips. Moreover, BroHealth has both English and French versions.

#### Telephone-Based Job Coaching

The rationale of including job coaching as part of the intervention is two-fold. First, previous research and our national survey of high-risk working men identified that a limitation of e-mental health programs is the lack of personal interaction [11,12,14]. Second, many RCTs on e-mental health programs suffered from high attrition rates [11,14,15], which is a major threat to the validity of the trials. One contributing factor that emerged from qualitative interviews is that e-mental health programs do not address issues that are relevant to the participants. Therefore, we proposed a combination of BroHealth and job coaching as an intervention arm.

The aim of coaching service is to help users set goals and discover answers to problems for themselves and to motivate users to take actions and engage their own solutions. Coaching is carried out by qualified people who work with clients to improve their effectiveness and performance. Job coaches are individuals who specialize in assisting individuals to learn and accurately carry out job duties. Job coaches provide one-on-one training tailored to the needs of the employee. The coach is well educated, skilled, and experienced in dealing with Human Resources advising, career counseling, goal setting, solution-based problem solving, and performance coaching. In the proposed trial, 5 coaches from different regions of Canada will be hired. Trial participants may access the coaching services in their region through an appointment booking system which is embedded in BroHealth. The coaches in Ontario, Quebec, and Atlantic Canada are bilingual (English and French). Participants will be eligible for one session per week. Each coaching session may last a maximum of 40 minutes.

### Outcomes and Measurements

#### Primary Outcome

The primary outcome is the 12-month risk proportion of MDE, which is the WHO’s Composite International Diagnostic Interview-Short Form (CIDI-SF) [16], will be administered to determine MDE in the past 12 months, based on the Diagnostic and Statistical Manual of Mental Disorders (DSM) criteria [17]. The Composite International Diagnostic Interview-Short Form for Major Depression (CIDI-SFMD) is a structured diagnostic interview for MDE, which has been repeatedly incorporated, without modification, into a series of national health surveys by Statistics Canada, starting in 1994. The CIDI-SFMD was developed from the full version of CIDI to provide a quick screen of MDE [16]. The WHO’s CIDI has been widely used in population-based mental health surveys in different countries. MDE represents a purported 90% predictive cut point for the CIDI-SFMD. Using the full CIDI diagnosis as “gold standard”, the sensitivity and specificity of the CIDI-SFMD was 89.6% and 93.9%, respectively [16].

#### Secondary Outcomes

Secondary outcome variables are changes in depression and anxiety scores, and absenteeism and presenteeism. Depression will be measured by the 9-item Patient Health Questionnaire (PHQ-9); anxiety will be measured by the 7-item Generalized Anxiety Disorder (GAD-7). The PHQ-9 is a widely used scale for assessing depressive symptoms [18], which scores each of the 9 symptoms from 0 (not at all) to 3 (nearly every day) [18]. A PHQ-9 score of greater than or equal to 10 has a sensitivity of 88% and a specificity of 88% for MDE [18]. The GAD-7 is widely used as a general screen for common symptoms of anxiety. The GAD-7 has good reliability and validity. A cut point was identified that optimizes sensitivity (89%) and specificity (82%) [19].

Absenteeism and presenteeism will be measured by the WHO’s Health Performance Questionnaire (HPQ) [20,21] and the Lam Employment Absence and Productivity Scale (LEAPS) [22]. The HPQ is a brief self-report questionnaire that obtains screening information about the prevalence and treatment of commonly occurring health problems, and information about 3 types of workplace consequences (sickness absence, presenteeism, and critical incidents). LEAPS is a validated scale for measuring occupational functioning for workers with depression.

#### Predicted Risk and Probability of MDE

Predicted risk and/or probability of MDE will be measured using the sex-specific risk prediction algorithm [13]. This risk prediction algorithm is to be used in individuals who do not have MDE. Based on exposure to a key set of risk factors including family history, current health status, childhood trauma, and ongoing life stressors, the algorithm can generate a person’s probability of having MDE in the next 4 years [13]. The algorithm was developed using the data from over 4700 Canadian men who were followed for 4 years [13].

#### Return on Investment

Based on the HPQ, lost work productivity will first be calculated. The financial value of lost work productivity will be calculated as:

Value = (A x B) x C

where A is the absolute hours of combined lost work productivity (from change over time from baseline period to follow-up period in absenteeism hours and poor job performance/presenteeism hours), B is the financial value of hour of employee compensation (wages + benefits value), and C is the economic productivity multiplier (1.25). An example is shown in Textbox 2.

**An example of the financial value of lost work productivity equation.**

Example

Per 1 month:

37 hours of lost productive time x $50 per hour = $1850

$1850 x 1.25 = $2312.50 total

This can be extrapolated to the entire year:

$2312.50 x 12 = $27,750 lost per person per year

However, this is a high estimate as normal workers are not 100% productive while at work, nor are they never absent from work. So this can also be adjusted to take into account normative employee levels of lost work time.

### Other Variables

Other variables include demographic and socioeconomic characteristics, occupation, employment status, job gradient, parental and marital strain [23], self-rated physical health, alcohol use, sleep disturbance, anger, quality of life, and mental health service use. Alcohol use will be measured by the 3-item Alcohol Use Disorders Identification Test (AUDIT-C). Sleep disturbance and anger will be assessed using the measures of Patient-Reported Outcome Measurement Information System (PROMIS) of the US Department of Health and Human Services. Quality of life will be assessed by the EuroQol five dimensions questionnaire (EQ-5D) which is a widely used instrument for quality of life [24]. Questions about mental health service use are adopted from Statistics Canada’s national health surveys.

### Recruitment and Screening

Recruitment and screening will be contracted to Leger that has access to the numbers of household landlines and mobile phones across the country. Recruitment will be conducted using the method of Random Digit Dialing (RDD). Once a potentially eligible participant is identified, the interviewer will confirm the participant’s age, working status, and access to the Internet for personal use, by asking “Do you have access to Internet for personal use, through computer or mobile device?” Then the CIDI-SFMD [16] will be administered. This is to assess MDE in the past 12 months and to ensure the participant is not experiencing a MDE at the time of interview.

If a participant has had MDE in the past 12 months, he will be asked “In the past 2 months or longer, has your mood been much improved or back to normal AND you DIDN’T have any symptom of (key phrases)?” Those who answer “no” will be excluded.

Following this, the sex-specific prediction algorithm for MDE will be administered. The algorithm for men contains 15 questions about personal and family history of MDE, current health status, childhood trauma, and recent negative life events [13]. With the input of the answers to these questions, the algorithm generates the participant’s probability of having MDE in the next 4 years. The original development analysis showed that a probability of 6.51% represented the top 20% of Canadian adult men in terms of the risk of having MDE [13]. In this study, those who have a risk less than 6.51% are considered being at low risk and will be excluded from the study.

### Baseline Assessment

If a person meets the eligibility criteria, the interviewer will administer demographic and occupational characteristics questions, PHQ-9 for depression, GAD-7 for anxiety, perceived risk of having MDE, the HPQ, LEAPS, self-rated physical health, alcohol use, sleep disturbance, anger, quality of life, and mental health service use.

### Online Assessment

To ensure the use of the Internet, participants who complete the baseline telephone assessment will be instructed to complete a short online survey as the last step of the baseline assessment and informed consent. The online survey will include questions about Internet use with the 12-item Job Content Questionnaire [25] used in the Statistics Canada survey to measure self-reported work stress, and consent to participation.

### Randomization

Participants who complete the baseline telephone and online surveys will be randomized into intervention and control groups. The randomization will be conducted and managed by the project coordinator affiliated with the project. To accomplish this, 1200 random numbers (between 0 and 1) will first be generated using Excel (“RAND()”) for 1200 study identification (ID) numbers, ranging from 1 to 1200. The 1200 random numbers will then be sorted in descending order and categorized into 3 equal groups with the ID numbers in the first group (n=400) allocated to the control group, the ID numbers in the second group (n=400) allocated to BroHealth only, and the ID numbers in the third group (n=400) allocated to BroHealth plus job coaching.

The Excel sheet with ID numbers and random numbers will be kept and printed out for randomization. For example, if a participant is the 3rd one to complete the online survey, determined by the date of completion, the project coordinator will search for ID #3 on the Excel sheet. If the random number associated with ID #3 is allocated to the second group, this participant will be assigned to the group that will receive BroHealth only.

The date of randomization will be documented. Any outcomes and/or changes that occur after the randomization will be counted to the groups, regardless of course of the BroHealth and job coaching interventions and if the interventions are used by the participants.

After randomization, the project coordinator will contact the participant by email. For those in the control group, the email will include a link to the men’s mental health page of the Movember Foundation website. For those in the BroHealth only group, the email will include all information to the controls, a link to BroHealth log-in page, and the user name and password, whereas for those in the BroHealth plus job coaching group, the email will include all information to the participants in other groups, and instruction to the coaching appointment booking system in BroHealth.

Afterwards, a package including a thank you letter, the Movember Foundation website link to men’s mental health, and Can $20 incentive as appreciation of their participation will be mailed to the participants.

### Follow-Up Assessments

The post-randomization assessments will be conducted at 6 and 12 months. At the 6- and 12-month assessments, information regarding demographic and occupational characteristics questions, including changes in marital status and employment, alcohol use, self-rated physical health, CIDI-SFMD, PHQ-9 for depression, GAD-7 for anxiety, WHO’s HPQ, sleep disturbance, anger, quality of life, and mental health service will be collected.

It is anticipated that time is needed to schedule follow-up interviews and there will be conflicts with scheduled interviews (eg, some participants may have to cancel a scheduled interview, or some do not respond to a scheduled phone call). Therefore, a 2-month interview window will be allowed for each follow-up interview.

To obtain in-depth information about how BroHealth affects men’s behaviors and risk profile, qualitative interviews will be conducted via telephone with 10% of the participants in the intervention groups at the end of the RCT (ie, after the 12-month assessment). The participants will represent those with diverse outcomes (clinical and occupational trajectories, and employment status changes). Qualitative interview data will yield in-depth data about the impacts of receiving BroHealth and job coaching on the outcomes. These data will be used to support and interpret the primary quantitative data base. The multiple forms of evidence generated through mixed methods designs are particularly useful for illuminating complex health issues [26].

### Data Management

The telephone interview firm will transfer password-protected baseline data to the principal investigator (PI) on a weekly basis. The 6- and 12-month follow-up assessments will be conducted at the telephone interview laboratory at the Mathison Centre for Mental Health Research & Education, University of Calgary. One month before the scheduled follow-up interviews, letters will be sent to the participants to remind them of the upcoming interview. An interview log will be developed for each participant to document interview time, schedule, call-back, contact information, and for the interviewer to make comments. All data will be kept confidential and stored on password-protected computers at the telephone interview laboratory and the PI's office, which are under 24/7 security surveillance.

Over the course of the trial, the PI and research staff will examine the data on a regular basis by running cross tabulation, frequency distribution, and estimation of means and proportions to ensure the quality of the data and identify missing values and potential outliers. If missing values and outliers are found, the records will be referred back to the interviewer for clarification or call-back.

After the 12-month interview, participants’ group status will be linked with interview data by study ID numbers. Over the study period, the investigators and the interviewers who conduct the follow-up interviews in Calgary will not have access to participants’ group status. The Leger interviewers, who conduct the baseline assessment and the project coordinator who manage the randomization process, will not be involved in follow-up interviews.

A sub-committee has been formed for this trial. The committee members will communicate via teleconference on a bi-monthly basis to review and discuss the progress, operational issues, final data analysis, and results interpretation.

### Statistical Analysis

All data analyses will be conducted using the most recent version of STATA. An Intention-to-treat (ITT) principle will be used in the analysis (eg, initial intervention assignment rather than intervention received will be used in the analysis).

For the primary objective, the proportions of MDE over 12 months will be estimated for each group and will be compared using chi-square tests between control versus intervention #1, and control versus intervention #2. Logistic regression modeling will be used to examine the effect of the intervention on the outcome, controlling for the effects of baseline confounders. Contrast analysis will be conducted to compare different intervention groups (intervention #1 vs intervention #2) as one of the secondary objectives.

For the secondary objectives, the distributions of the changes in the depression and anxiety scores at 6 and 12 months in the intervention and control groups will be examined. The means of the changes between the intervention and control groups will be compared using *t* tests.

If the assumption of normal distribution is violated, transformation or non-parametric testing will be used. Analysis of variance (ANOVA) will be conducted to examine between group differences (separately for the 6- and 12-month assessments), controlling for the effects of baseline characteristics. Repeated-measures mixed models will be used to examine intervention effect (time x intervention), controlling for the effects of baseline characteristics. The analyses will be repeated for absenteeism and presenteeism (secondary objectives).

An economic evaluation will quantify the costs associated with work productivity loss and absence from work. Self-report measures of work performance on the HPQ can be converted into a percentage of work productivity hours and total hours of work absence. The total combined hours of work performance can then be assigned a dollar value based on consulting industry standard practices for estimating the value of an hour of work absence/productivity, typically a multiplier (eg, 1.5 or 2.0) of the employee hourly compensation rate [27,28]. The hourly compensation rate (wages and benefits combined dollar value) will be estimated for each respondent based on the mid-point of the income category level reported on the baseline assessment. The analysis will then compare the intervention versus control groups for net changes over time to determine if the intervention has a relatively more positive outcome in average work performance over the study period on a per participant basis. This dollarized outcome can then be compared to the cost of providing the intervention to yield an overall cost-benefit or return on investment test [29].

### Sample Size Calculation

We propose recruiting 400 participants for each group (N=1200) at baseline. Imamura and colleagues assessed the effectiveness of integrative CBT (iCBT) in reducing the risk of MDE in a RCT [30]. Participants (N=762) were randomized into control and intervention group. Over 12 months, 0.8% and 3.9% of participants in the intervention and control group developed MDE, respectively. Based on these proportions, 374 participants in each arm are needed to detect the difference at a power of 0.80 and alpha level of.05. The sample size was calculated using statistical software STATA 14.0 (syntax: power two proportions 0.008 0.039) [31]. This is consistent with the average number of participants in the previous RCTs [32]. To offset attrition, we plan to provide a Can $20 incentive for participating in an assessment in appreciation of the participants’ time and efforts.

## Results

This study was approved by the Conjoint Health Research Ethics Review Board of the University of Calgary (Ethics ID: REB14-2365). The trial is funded by a team grant from the Movember Foundation, a global charity for men’s health. BroHealth was developed at Digital Emergency Medicine, University of British Columbia, and the usability testing is complete. It is anticipated that the RCT will be completed by 2018.

## Discussion

### Principal Findings

BroHealth is novel in several ways. First, BroHealth focuses on early prevention of major depression, rather than providing treatment to those who already have MDE. The outcomes include not only reduced risk of MDE, but also improved work functioning, productivity, and return on investment. Second, BroHealth was developed through a gender lens, focusing on the working men who are at high risk of having MDE. Third, the development of BroHealth was informed by men’s preferences of design features of e-mental health programs, through a national survey of the target population. To this extent, this approach enhances the acceptability of BroHealth by working men who are at high risk of MDE.

### Limitations

BroHealth was designed to be used by male workers who have access to the Internet. Men who don’t have Internet access for personal use will not be included. Some elements of BroHealth were based upon existing programs. For example, the CBT program of BroHealth was adapted from the abbreviated version of the UK Living Life to the Full (LLTTF), and the mindfulness relaxation program was developed by University Health Network in Ontario. However, the French materials for the mindfulness relaxation program were not available. This may affect the effectiveness of BroHealth in French speaking participants. We will examine this potential impact by conducting the proposed analyses in English and French speaking participants separately. The incidence of MDE will be measured by CIDI-SF for MDE, which is a fully structured diagnostic interview, rather than by physician diagnosis. Therefore, misclassification of diagnosis is possible.

### Conclusion

MDE is a prevalent mental health condition in working men. Because of the stigma against depression and gender/social norms, men who are at high risk of MDE are reluctant to seek help and are lack of skills of self-management. BroHealth is a tool designed to overcome these gaps. The proposed RCT will provide solid evidence about the effectiveness and return on investment of this product.

## Abbreviations

CBT: cognitive behavioral therapy
CIDI: Composite International Diagnostic Interview-Short Form
CIDI-SFMD: Composite International Diagnostic Interview-Short Form for Major Depression
e-mental health: electronic mental health
GAD-7: Generalized Anxiety Disorder
HPQ: Health Performance Questionnaire
ID: identification
LEAPS: Lam Employment Absence and Productivity Scale
MDE: major depression
PHQ-9: Patient Health Questionnaire
PI: principal investigator
RCT: randomized controlled trial
WHO: World Health Organization
